# Analysis of the Relationship between Obesity Factors and Health-Related Physical Fitness Factors among People with Intellectual Disabilities in South Korea

**DOI:** 10.3390/ijerph18126533

**Published:** 2021-06-17

**Authors:** Guyeol Jeong, Buongo Chun

**Affiliations:** 1 Department of Social Physical Education, College of Humanities and Arts, Sunchon National University, Sunchon 57922, Korea; zzzinsang@hanmail.net; 2 Graduate School of Physical Education, College of Arts and Physical Education, Myongji University, Yongin 17058, Korea

**Keywords:** people with intellectual disability, overweight, obesity, health-related physical fitness factor, muscular endurance, relationship

## Abstract

Little is known about the relationship between health-related physical fitness factors and obesity-related factors in individuals with intellectual disabilities. We investigated the prevalence of obesity and the relationship between obesity-related factors and health-related physical fitness factors among people with intellectual disabilities in South Korea to identify the physical fitness factors that influence the degree of obesity. For obesity-related factors, we measured body composition (height, weight, body mass index—BMI, and body fat percentage) of 108 subjects with intellectual disabilities (mean ± standard deviation; age = 24.4 ± 8.45 years). For health-related physical fitness factors, we measured muscular strength, muscular endurance, and flexibility. There was a significant negative correlation between muscular endurance and obesity factors (BMI, *r* = −0.306, *p* < 0.01; body fat percentage, *r* = −0.402, *p* < *0*.01). Further, the prevalence of obesity (34.3%) and being overweight (17.6%) among people with intellectual disabilities was high in South Korea. In addition, muscular endurance was found to have a significant effect on the obesity level (β = −0.239, *p* < 0.000). This suggests that a program that considers muscular endurance should be prioritized when implementing an exercise intervention strategy for the treatment of overweight and obesity among people with intellectual disabilities.

## 1. Introduction

Obesity is a complex chronic disease characterized by excessive accumulation of body fat. It has been declared a global pandemic by the World Health Organization (WHO) [1]. Its prevalence is high among children, adolescents, and adults in developed countries [2]. Obesity is considered a dangerous disease because it causes or worsens many health problems independently or in connection with other diseases. The WHO defines obesity as body mass index (weight—kg divided by the square of height—m^2^; body mass index—BMI) of over 30 kg/m^2^ [3,4]. Among Asians, obesity is defined as a BMI of ≥25 kg/m^2^ [5]. BMI is a person’s weight in kilograms divided by the square of height in meters. BMI is an inexpensive and easy screening method for weight categories, such as underweight, healthy weight, overweight, and obesity. Obesity has negative effects on health, such as type 2 diabetes, hypertension, cardiovascular disease, and premature death. It is also highly related to social and psychological problems as well as a social and economic burden [6,7,8,9]. Since such obesity-related costs impose a large economic burden on society [10], rapid intervention and treatment for obesity are required.

In particular, it has been reported that the prevalence of obesity is higher among people with intellectual disabilities than among the general population [11,12,13]. According to a study on people with intellectual disabilities in Australia, the prevalence of obesity (23.8%) and being overweight (22.5%) was higher in this population than that in the general population [12]. In the UK, the prevalence of obesity and being overweight among adults with intellectual disabilities is 20.7% and 28%, respectively [11]. This indicates that obesity is a more serious problem among people with intellectual disabilities than among the general population [14,15].

On the other hand, it has been reported that the number of people with intellectual disabilities is the highest in low- and middle-income countries [16]. Currently, there are 2.59 million people with disabilities in South Korea, accounting for 5% of the total population, of which 212,936 have intellectual disabilities. The proportion of people with intellectual disabilities among the population with disabilities consistently increased from 6.9% in 2009 to 8.2% in 2015, to 9.0% in 2018 [17]. Although it may seem that the prevalence of being overweight and obese among people with intellectual disabilities is higher in South Korea than in Western countries due to social and environmental problems, studies examining such prevalence are limited.

Obesity is caused by a variety of factors, including environmental and genetic factors [18]. In particular, it has been reported that decreased physical activity, decreased physical fitness, inadequate nutritional intake, and a sedentary lifestyle are risk factors of obesity [19]. In this regard, people with intellectual disabilities are limited in their physical activities due to limitations in language development, cognitive function, and cognitive impairment [20]. It has also been reported that many of them lead a sedentary lifestyle [21]. In addition, as they have a stronger desire for food intake than the general population, the prevalence of obesity is also higher [22]. Their lack of physical activity leads to a decline in their fitness level, which, in turn, causes various diseases such as obesity, hypertension, type 2 diabetes, and cardiovascular disease, thereby decreasing the health of people with intellectual disabilities [23]. Previous studies have shown that people with low fitness levels have a higher risk of becoming overweight or obese than those with high fitness levels [24,25]. Other studies have also shown that obese or overweight individuals have lower physical fitness levels than do people with a normal weight [26], and that physical fitness and body fat are negatively correlated [27].

It can be assumed that higher physical fitness levels among people with intellectual disabilities are associated with better body composition. However, little is known about the relationship between health-related physical fitness factors and obesity-related factors in this population. In addition, while the prevalence of intellectual disabilities and obesity is increasing in middle-income countries such as South Korea, no survey data have been reported on the obesity rates of teenagers to adults in their 40 s or the relationship between obesity and health-related physical fitness factors. Therefore, we aimed to investigate the prevalence of obesity among people with intellectual disabilities in South Korea and examine the relationship between health-related physical fitness and obesity-related factors. Elucidating the physical factors associated with obesity in people with intellectual disabilities could lead to the development of new intervention programs to promote a healthier lifestyle in this population. Our results can help researchers, clinical experts, and trainers develop intervention strategies for the treatment of obesity and further motivate policymakers and decision makers to prioritize the treatment of people with intellectual disabilities.

## 2. Materials and Methods

### 2.1. Subjects

In total, 108 individuals with intellectual disabilities (75 men and 33 women) residing in City G in South Korea were included in this study. They were selected from people with intellectual disabilities who visited the Physical Fitness Certification Center for the Disabled in City G (https://nfa.koreanpc.kr/front/centerpop/bs/boardList.do?center_cd=002&board_seq=14&type=popup, accessed on 23 January 2019). The inclusion criteria for subjects in this study are as follows: (1) participants, parents, and/or legal guardians have signed prior consent before participation; (2) people who can walk without help; (3) people without motor impairment. The exclusion criteria for the subjects in this study are as follows: (1) people who are prohibited from exercising; (2) persons with a physical disability who are unable to perform physical activities. After we provided a thorough explanation of the purpose of our study and the measurement method to the visitors and their guardians in accordance with the Ethical Principles of the Declaration of Helsinki, we selected subjects among those who gave consent. In addition, for the sake of consistency, measurement and evaluation experts at the Physical Fitness Certification Center for the Disabled performed the measurements. Table 1 shows the general characteristics of the participants.

### 2.2. Measurement

The health-related physical fitness of people with intellectual disabilities who participated in this study was measured using measuring instruments available at the Physical Fitness Certification Center for the Disabled in City G. As for the measurement criteria, body composition (height, weight, BMI, and body fat percentage), muscular strength, muscular endurance, and flexibility were measured.

#### Body Composition

The height (cm), weight (kg), BMI, and body fat percentage of subjects with intellectual disabilities were measured using an automatic height–weight scale (BSM 330, Inbody, Seoul, Republic of Korea) and bioelectric impedance analysis (InBody 770, InBody, Seoul, Republic of Korea) [28]. BMI was calculated by dividing the weight in kilograms by the height in meters. Participants were classified as normal weight (BMI < 23), overweight (BMI ≥ 23), and obese (BMI ≥ 25) [5]. Obesity was defined as BMI ≥ 25 kg/m^2^ according to the Asia Pacific standards of the WHO guidelines [5]. The method of measuring the body composition required the subjects to wear light clothing (exercise clothing) and to place the soles of their bare feet evenly on the footing. Standing in an upright position facing the front, holding the grip with both hands, and opening the gap between the armpits. When the machine scans the body, it is measured while keeping the body immobile [29]. For accurate measurements, we asked the subjects to change into clothing that was as simple as possible, to remove any substances attached to the body, and to take measurements an hour after emptying their bowels.

### 2.3. Health-Related Physical Fitness

Health-related physical fitness was measured using variables included in the physical fitness criteria for people with intellectual disabilities at the Physical Fitness Certification Center for the Disabled. Before measuring physical fitness, simple warm-up exercises and stretching were performed for approximately five minutes with the help of measurement and evaluation experts. The following sections describe the measurement methods for each criterion.

#### 2.3.1. Muscular Strength

Muscular strength was measured by using a grip strength dynamometer (BS-HG, Inbody, Seoul, Korea) [30,31,32]. For the measurement method, the subject needed to stand shoulder-width apart, and hold the grip while the body and arms are stretched out, about 15 degrees apart. The measurement was made with a maximum pull of over two to three seconds from the start of the measurement. In cases where people with intellectual disabilities could not hold the instrument firmly, assistants helped them to grip and exert maximum force and maintain it for five seconds. Muscular strength was measured twice and was recorded in units of 0.1 kg of the highest value.

#### 2.3.2. Muscular Endurance

The sit-up test was used to measure muscular endurance (BS-SU, Inbody, Seoul, Republic of Korea) [33,34]. Prior to the measurement of muscular endurance, we asked subjects to do the following: (1) lie on their head and back, on the mattress; (2) place their knees at a 45° angle and make a gap between them, such that it fits into a fist; (3) perform sit-ups for a minute while clasping both hands behind their head. The two elbows were to touch the knees and then return to their initial positions once. The unit of measurement for muscular endurance was the number of repetitions per 30 s. In order to help people with intellectual disabilities understand the procedure, we let them practice two to three times with the help of assistants and we informed them of the number of repetitions during the measurement.

#### 2.3.3. Flexibility

Flexibility was measured using sit-and-reach exercises by a buckling flexion meter (BS-FF, Inbody, Seoul, Korea) [34,35]. Subjects were asked to take their shoes off and straighten their legs so that both heels were in close contact with the measurement instrument, then overlap their hands, and extend their arms forward. The upper body was bent as far as possible to push out the ruler. We took the measurement twice and selected the highest value. If a subject could not stretch their knees because of having a low cognitive ability due to an intellectual disability, an assistant pressed the knee of the subject to prevent it from bending and provided a demonstration to prevent the pushing of the lateral flexometer measurement plate due to recoil.

### 2.4. Data Analysis

We calculated the mean (M) and standard deviation (SD) for each criterion using the Statistical Package for the Social Sciences version 22 (IBM Corp., Armonk, NY, USA). A frequency analysis was performed to explain the general characteristics of the subjects. Further, a cross-analysis was conducted to examine the ratio of obesity and being overweight according to the sex and age group. Pearson’s correlation analysis was conducted to examine the relationship between obesity-related factors (BMI, body fat percentage) and health-related physical fitness factors (muscular strength, muscular endurance, and flexibility). Multiple regression analysis was conducted to examine which variables affected the degree of obesity. The statistical significance level was set at *p* < 0.05.

## 3. Results

### 3.1. Analysis of Obesity Severity According to the Sex of People with Intellectual Disabilities

**Figure 1:**
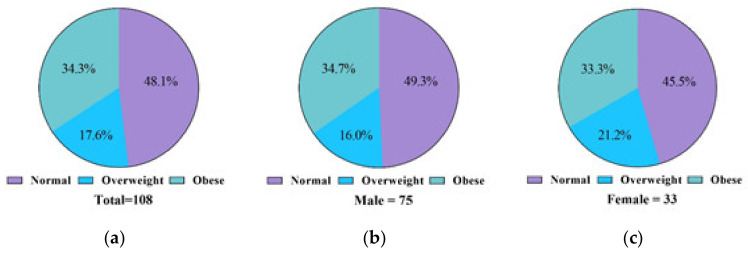
Percentages of Koreans in this study with intellectual disabilities who have a normal weight, are overweight, and obese: (**a**) percentage of overweight and obese (total subjects (*N* = 108)); (**b**) percentage of overweight and obese male subjects (*n* = 75); (**c**) percentage of overweight and obese female subjects (*n* = 33).

The analysis of obesity severity according to sex among people with intellectual disabilities is shown in Figure 1. The prevalence of obesity (according to BMI) among people with intellectual disabilities was 34.3%. There was no significant difference between men (34.7%) and women (33.3%). According to BMI, the ratio of being overweight among people with intellectual disabilities was 17.6% (19 people). Although female subjects (21.2%; 7 people) showed a higher ratio than male subjects (16.0%; 12 people), there was no significant difference (x**^2^** = 0.0.437; *p* = 0.804). According to BMI, the proportion of people with intellectual disabilities that belong to the normal weight range was 48.1% (52 people). There was no significant difference between male (49.3%; 37 people) and female (45.5%; 15 people) subjects.

### 3.2. Analysis of Obesity Severity According to Age among People with Intellectual Disabilities

The analysis of obesity severity according to the age group of people with intellectual disabilities is summarized in Table 2. The prevalence of obesity tended to increase with age: the prevalence was 19.4% in people aged 10–19 years old (6 people), 35.7% (20 people) in subjects aged 20–29 years old, 38.5% (5 people) in subjects aged 30–39 years old, and 75% (6 people) in subjects aged 40–49 years old. However, there was no significant difference between age groups. The proportion of overweight subjects was 12.9% (4 people) in the 10–19 age group, 19.6% (11 people) in the 20–29 age group, 23.1% (3 people) in the 30–39 age group, and 12.5% (1 person) in the 40–49 age group. The proportion of subjects that belonged to the normal weight range was 67.7% (21 people) in the 10–19 age group, 44.6% (25 people) in the 20–29 age group, 38.5% (5 people) in the 30–39 age group, and 12.5% (1 person) in the 40–49 age group. There was no significant difference between the age groups. In addition, the basic characteristics (age, height, and weight) of the participants, obesity-related factors (BMI and % body fat), and health-related fitness factors (sit-up, sit and reach, and grip strength) are shown in Table 3.

### 3.3. Correlation between Factors among People with Intellectual Disabilities

Table 4 shows the results of the correlation analysis between age, body composition, and health-related physical fitness factors of people with intellectual disabilities. Weight showed positive correlations with age (r = 0.309, *p* < 0.01) and height (r = 0.459, *p* < 0.01); BMI showed positive correlations with age (r = 0.275, *p* < 0.01) and weight (r = 0.873, *p* < 0.01).

**Figure 2:**
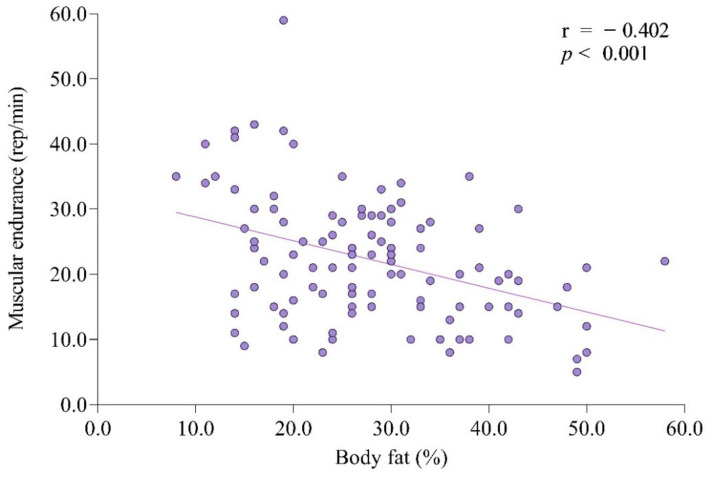
The relationship between body fat and muscular endurance (sit-ups) among Korean participants with intellectual disability. Body fat percentage is negatively correlated with sit-ups (*r* = −0.402, *p* < 0.001).

Body fat percentage was negatively correlated with height (*r* = −0.263, *p* < 0.01) and positively correlated with weight (*r* = 0.560, *p* < 0.01) and BMI (*r* = 0.789, *p* < 0.01). Sit-ups showed a negative correlation with age (*r* = −0.282, *p* < 0.01), weight (*r* = −0.222, *p* < 0.05), BMI (*r* = −0.306, *p* < 0.01), and body fat percentage (*r* = −0.402, *p* < 0.01) (Figure 2). Sit and reach was positively correlated with sit-ups (*r* = 0.285, *p* < 0.01). Grip strength was positively correlated with age (*r* = 0.270, *p* < 0.01), height (*r* = 386, *p* < 0.01), weight (*r* = 0.235, *p* < 0.05), sit-ups (*r* = 0.318, *p* < 0.01), and sit and reach (*r* = 0.312, *p* < 0.01).

### 3.4. Factors Affecting the Body Fat Percentage of People with Intellectual Disabilities

To examine the factors associated with body fat percentage, which had the highest significant correlation with the obesity level of people with intellectual disabilities, we performed multiple regression analysis (Table 5). When we analyzed the effects of height, weight, and sit-up factors (which showed significant correlations with body fat percentage) on body fat percentage, we found that their explanatory power (R2) was 72.5% and that the results were statistically significant (F = 90.361, *p* < *0*.001). In addition, we tested the significance of each factor and found that height (*p* < 0.001), weight (*p* < 0.001), and sit-ups (*p* < 0.001) had significant effects on body fat percentage. In order to determine the importance of the factors influencing body fat percentage, we compared the standardized regression coefficients (β values) and found that height (β = −0.735), weight (β = 0.591), and sit-ups (β = −0.239) had an effect on body fat percentage in decreasing order.

## 4. Discussion

Our study aimed to investigate the prevalence of obesity and physical fitness factors affecting obesity among people with intellectual disabilities in South Korea. Our results show a high prevalence of overweight and obesity among people with intellectual disabilities in South Korea. The prevalence of overweight and obesity among people with intellectual disabilities was similar between male and female subjects. This shows a tendency to increase with age. In addition, there was a significant correlation between health-related physical fitness factors (muscular endurance) and obesity factors (BMI and body fat percentage) among people with intellectual disabilities in South Korea. Moreover, the muscular endurance factor had a significant effect on the body fat percentage (Figure 2).

Obesity is a negative body change that degrades the quality of human life, regardless of the presence or absence of a disability, and requires continuous and systematic management. As people with intellectual disabilities tend to have low levels of physical activity and indulge in excessive nutritional intake, they are prone to having an unbalanced body, being overweight, and having impaired body functions, which eventually leads to obesity [28].

The prevalence rates of overweight (17.6%) and obesity (34.3%) among people with intellectual disabilities in this study were similar to those of other countries. For example, in an Australian study of 206 subjects, approximately 22.5% of subjects were obese, and 23.8% were overweight [12]. A study in the UK also reported that the prevalence rates of obesity and overweight were 20.7% and 28.0%, respectively, whereas subjects that fell in the normal range of weight accounted for 32.7% [11]; a study by Hsieh et al. [36] on people with intellectual disabilities in the United States reported prevalence rates of 28.9% for overweight and 38.3% for obesity. The prevalence of overweight and obesity is higher among people with intellectual disabilities than in the general population [12,13,37,38]. This may be because people with intellectual disabilities have decreased income levels in an environment where they have difficulty engaging in economic activities, which leads to a lack of opportunities to participate in physical activities and exercise programs [20,39]. At this rate, obesity among people with intellectual disabilities is expected to increase more rapidly in the future, thereby increasing the morbidity rate as well. Therefore, it is necessary to pay closer attention to obesity treatment interventions for people with intellectual disabilities.

In our study, there was no significant difference between the ratio of being overweight (male 16.0% vs. female 21.2%) and the prevalence of obesity (male 34.7% vs. female 33.3%) according to sex. This is supported by a meta-analysis on adults with intellectual disabilities, which showed no difference in sedentary behavior between genders [40]. However, it is necessary to prioritize interventions for women with intellectual disabilities, given that the following studies: the study in [40] showed a significantly lower physical activity level in female, compared with male, subjects with intellectual disabilities, and Hsieh et al. [36] suggested that women have a higher risk of becoming severely obese than men with intellectual disabilities.

It has been reported that overweight and obese people have lower physical fitness than those with normal weight. However, to the best of our knowledge, few studies have analyzed the relationship between health-related physical fitness factors and obesity factors among people with intellectual disabilities. Our results showed an inverse relationship between muscular endurance and BMI as well as the body fat percentage of people with intellectual disabilities (BMI, *r* = −0.306; body fat percentage, *r* = −0.402). In addition, through multiple regression analysis, we found that muscular endurance had a significant effect on body fat percentage among people with intellectual disabilities (β = −0.218, *p* = 0.000).

Although it is difficult to make a direct comparison, several studies on people without disabilities have shown a negative relationship between obesity factors and physical fitness [26,27,41,42]. A previous study by Fogelholm et al. [27] in normal adolescents showed a strong negative relationship between muscular endurance (sit-ups) and being overweight. A similar result was found in a study by Lee and Oh [43], who examined male Korean adolescent subjects without disabilities (*n* = 3047) and found that muscular endurance is a significant factor affecting obesity. These results suggest that exercise programs that improve muscular endurance should be considered during interventions for people with intellectual disabilities who are overweight and obese.

Muscular endurance refers to the ability to use the muscles continuously for a certain period of time [44] and can be improved by strength training. The exercise method that can improve muscular endurance in a population with a low physical fitness level is as follows: perform 10–15 repetitions of an exercise with an intensity of one repetition maximum of 40–50% or less, one to two sets per muscle area, and with short breaks between each set [44,45,46]. Although aerobic exercise for cardiorespiratory fitness may be important for people with intellectual disabilities, it may be wise to focus instead on improving muscular endurance, because low muscular endurance can limit aerobic exercise performance [47].

Meanwhile, muscular strength and flexibility were not shown to significantly affect obesity-related factors in intellectually disabled people. These results are similar to those of previous studies, which found a static correlation between weight and muscle strength [48,49]. Muscular strength is defined as the ability of a muscle to exert maximum contractile force at once, against resistance [50,51,52]. Muscular strength can be important for everyday life, in intellectually disabled people [53,54]. However, our results suggest that muscular strength is the next consideration in exercise programs to mediate obesity. Regarding flexibility, many previous studies have reported no significant association with obesity [55,56,57]. Although flexibility does not significantly affect obesity factors, it is believed that some participation in exercise could help in the daily lives of intellectually disabled people. This is because lack of flexibility is associated with musculoskeletal damage and back pain [55,58].

Given the small sample size of our study, it is difficult to generalize our results. Future research should include a larger number of subjects and investigate the relationship and determinants of various obesity-related physical fitness factors in order to promote health among people with intellectual disabilities.

## 5. Conclusions

Our results show an inverse relationship between muscular endurance and obesity factors among people with intellectual disabilities in South Korea. In addition, muscular endurance factors were found to have a significant effect on the body fat percentage in this population. This suggests that a program that takes muscular endurance into account should be considered when implementing an exercise intervention strategy for the treatment of overweight and obesity among people with intellectual disabilities. In addition, we confirmed, though with a small sample size, that the prevalence of overweight and obesity in Korean people with intellectual disability may be relatively high. It will be necessary to determine the continued prevalence of obesity by adding the number of cases in the future. Since an increase in the obesity rate leads to various diseases and is an important factor that lowers the quality of life of people with disabilities, developing an effective management method is needed. Our results can help researchers, clinical experts, and trainers develop intervention strategies for the treatment of obesity and further motivate policymakers and decision makers to prioritize the treatment of people with intellectual disabilities.

## Figures and Tables

**Table 1:** Subject characteristics.

Variables	Total (*N* = 108)	Male (*n* = 75)	Female (*n* = 33)
Age	24.40 ± 8.45	24.99 ± 7.92	23.06 ± 9.56
Height (cm)	163.97 ± 9.48	166.88 ± 8.66	157.34 ± 7.87
Weight (kg)	63.67 ± 14.61	64.38 ± 14.71	62.06 ± 14.48
BMI (kg/m^2^)	23.62 ± 4.97	22.98 ± 4.43	25.08 ± 5.82
% Body fat (%)	27.94 ± 10.46	24.24 ± 8.41	36.62 ± 9.71

Data are presented as the mean ± standard deviation. BMI, body mass index.

**Table 2:** Frequency analysis of obesity level by age group in participants with intellectual disabilities.

Variables	Age 10–19 (*n* = 31)	Age 20–29 (*n* = 56)	Age 30–39 (*n* = 13)	Age 40–49 (*n* = 8)	x^2^
Normal	21 (67.7%)	25 (44.6%)	5 (38.5%)	1 (12.5%)	11.829 (*p* = 0.066)
Overweight	4 (12.9%)	11 (19.6%)	3 (23.1%)	1 (12.5%)	
Obese	6 (19.4%)	20 (35.7%)	5 (38.5%)	6 (75.0%)	

Data are presented as frequency (percentage); x^2^ = Chi-square.

**Table 3:** Descriptive statistics for the whole study cohort (*n* = 108).

Variables	*n*	Mean	SD	SE	Min	Max	Range	95%CI
Age	108	24.38	8.49	0.82	12.00	46.00	34.00	(22.76; 26.01)
Height (cm)	108	164.13	9.36	0.91	135.50	187.20	51.70	(162.34; 165.93)
Weight (kg)	108	63.59	14.66	1.42	33.20	108.90	75.70	(60.78; 66.40)
BMI (kg/m^2^)	108	23.53	4.89	0.47	15.22	39.33	24.11	(22.59; 24.46)
% Body fat (%)	108	27.94	4.89	1.01	7.60	58.30	50.70	(22.59; 24.46)
Sit-up	108	22.22	9.52	0.92	5.00	59.00	54.00	(20.40; 24.05)
Sit and reach	108	0.21	11.94	1.15	−30.00	23.20	53.20	(−2.08; 2.50)
Grip strength	108	20.66	8.73	0.84	6.40	43.30	36.90	(18.99; 22.33)

SD, standard deviation; SE, standard error; Min, minimum; Max, maximum; CI, confidence interval; BMI, body mass index.

**Table 4:** Correlation analysis among age, body composition, and health-related physical fitness in people with intellectual disabilities.

Variables	Age	Height	Weight	BMI	Body Fat Percentage	Sit-Up	Sit and Reach	Grip Strength
Age	1							
Height	0.160	1						
Weight	0.309 **	0.459 **	1					
BMI	0.275 **	−0.021	0.873 **	1				
Body fat percentage	0.162	−0.263 **	0.560 **	0.789 **	1			
Sit-up	−0.282 **	0.045	−0.222 *	−0.306 **	−0.402 **	1		
Sit and reach	−0.014	−0.132	−0.162	−0.110	−0.120	0.285 **	1	
Grip strength	0.270 **	0.386 **	0.235 *	0.028	−0.173	0.318 **	0.312 **	1

BMI, body mass index; *, *p* > 0.05; **, *p* > 0.01.

**Table 5:** Factors affecting body fat percentage.

Variables	Non-Standardized Coefficients		Standardized Coefficients	*t*	*p*	VIF
	B	SE	β			
Constant	116.28	9.833		11.825	0.000	
Height	−0.735	0.066	−0.658	−11.078	0.000	1.319
Weight	0.591	0.043	0.828	13.616	0.000	1.383
Sit-up	−0.239	0.059	−0.218	−4.075	0.000	1.067

R = 0.851, R^2^ = 0.725, F = 90.361, *p* = 0.000; B, unstandardized beta coefficient; SE, standard error; β, standarded beta coefficient; VIF, variance inflation factor.

## Data Availability

The data that support the findings of this study are available from the corresponding author, [B.C.], upon reasonable request.

## References

[1] WHO (2000). Obesity: Preventing and Managing the Global Epidemic. Report of a WHO Consultation.

[2] Wang Y., Lobstein T. (2006). Worldwide trends in childhood overweight and obesity. Int. J. Pediatr. Obes..

[3] Kopelman P.G. (1994). Investigation of obesity. Clin. Endocrinol..

[4] Kopelman P.G. (2000). Obesity as a medical problem. Nature.

[5] Seo M.H., Lee W.-Y., Kim S.S., Kang J.-H., Kang J.-H., Kim K.K., Kim B.-Y., Kim Y.-H., Kim W.-J., Kim E.M. (2019). 2018 Korean Society for the Study of Obesity Guideline for the Management of Obesity in Korea. Obes. Metab. Syndr..

[6] Dixon J.B. (2010). The Effect of Obesity on health outcomes. Mol. Cell. Endocrinol..

[7] Dong B., Wang Z., Wang H.-J., Ma J. (2015). The association between resting heart rate and blood pressure among children and adolescents with different waist circumferences. Eur. J. Pediatr..

[8] Finkelstein E.A., daCosta DiBonaventura M., Burgess S.M., Hale B.C. (2010). The costs of obesity in the workplace. J. Occup. Environ. Med..

[9] Saner C., Simonetti G.D., Wühl E., Mullis P.E., Janner M. (2016). Circadian and ultradian cardiovascular rhythmicity in obese children. Eur. J. Pediatr..

[10] Wang Y., Beydoun M.A., Liang L., Caballero B., Kumanyika S.K. (2008). Will All americans become overweight or obese? Estimating the progression and cost of the US obesity epidemic. Obesity.

[11] Bhaumik S., Watson J.M., Thorp C.F., Tyrer F., McGrother C.W. (2008). Body mass index in adults with intellectual disability: Distribution, associations and service implications: A population-based prevalence study. J. Intellect. Disabil. Res..

[12] Krause S., Ware R., McPherson L., Lennox N., O’Callaghan M. (2016). Obesity in adolescents with intellectual disability: Prevalence and associated characteristics. Obes. Res. Clin. Pract..

[13] Segal M., Eliasziw M., Phillips S., Bandini L., Curtin C., Kral T.V.E., Sherwood N.E., Sikich L., Stanish H., Must A. (2016). Intellectual disability is associated with increased risk for obesity in a nationally representative sample of U.S. children. Disabil. Health J..

[14] De Winter C.F., Bastiaanse L.P., Hilgenkamp T.I.M., Evenhuis H.M., Echteld M.A. (2012). Overweight and obesity in older people with intellectual disability. Res. Dev. Disabil..

[15] Yamaki K. (2005). Body Weight Status among adults with intellectual disability in the community. Ment. Retard..

[16] Maulik P.K., Mascarenhas M.N., Mathers C.D., Dua T., Saxena S. (2011). Prevalence of intellectual disability: A meta-analysis of population-based studies. Res. Dev. Disabil..

[17] Korea Ministry of Health and Welfare Disabled Person Present Condition. https://www.index.go.kr/potal/main/EachDtlPageDetail.do?idx_cd=2768.

[18] Qi L., Cho Y.A. (2008). Gene-environment interaction and obesity. Nutr. Rev..

[19] Pronk N.P., Anderson L.H., Crain A.L., Martinson B.C., O’Connor P.J., Sherwood N.E., Whitebird R.R. (2004). Meeting recommendations for multiple healthy lifestyle factors. prevalence, clustering, and predictors among adolescent, adult, and senior health plan members. Am. J. Prev. Med..

[20] Salaun L., Berthouze-Aranda S.E. (2012). Physical fitness and fatness in adolescents with intellectual disabilities. J. Appl. Res. Intellect. Disabil..

[21] Oviedo G.R., Travier N., Guerra-Balic M. (2017). Sedentary and physical activity patterns in adults with intellectual disability. Int. J. Environ. Res. Public Health.

[22] Kolset S.O., Nordstrøm M., Hope S., Retterstøl K., Iversen P.O. (2018). Securing rights and nutritional health for persons with intellectual disabilities—A pressing challenge. Food Nutr. Res..

[23] Draheim C.C., Williams D.P., McCubbin J.A. (2002). Prevalence of physical inactivity and recommended physical activity in community-based adults with mental retardation. Ment. Retard..

[24] He Q., Wong T., Du L., Jiang Z., Yu T.I., Qiu H., Gao Y., Liu W., Wu J. (2011). Physical activity, cardiorespiratory fitness, and obesity among Chinese children. Prev. Med..

[25] Aires L., Silva P., Silva G., Santos M.P., Ribeiro J.C., Mota J. (2010). Intensity of physical activity, cardiorespiratory fitness, and body mass index in youth. J. Phys. Act. Health.

[26] Fiori F., Bravo G., Parpinel M., Messina G., Malavolta R., Lazzer S. (2020). Relationship between body mass index and physical fitness in Italian prepubertal schoolchildren. PLoS ONE.

[27] Fogelholm M., Stigman S., Huisman T., Metsämuuronen J. (2008). Physical fitness in adolescents with normal weight and overweight. Scand. J. Med. Sci. Sports.

[28] McLester C.N., Nickerson B.S., Kliszczewicz B.M., McLester J.R. (2020). Reliability and agreement of various inbody body composition analyzers as compared to dual-energy X-ray absorptiometry in healthy men and women. J. Clin. Densitom..

[29] Lee S.Y., Ahn S., Kim Y.J., Ji M.J., Kim K.M., Choi S.H., Jang H.C., Lim S. (2018). Comparison between dual-energy X-ray absorptiometry and bioelectrical impedance analyses for accuracy in measuring whole body muscle mass and appendicular skeletal muscle mass. Nutrients.

[30] Massy-Westropp N.M., Gill T.K., Taylor A.W., Bohannon R.W., Hill C.L. (2011). Hand grip strength: Age and gender stratified normative data in a population-based study. BMC Res. Notes.

[31] Wang Y.C., Bohannon R.W., Li X., Sindhu B., Kapellusch J. (2018). Hand-grip strength: Normative reference values and equations for individuals 18 to 85 years of age residing in the United States. J. Orthop. Sports Phys. Ther..

[32] Kong Z., Sze T.M., Yu J.J., Loprinzi P.D., Xiao T., Yeung A.S., Li C., Zhang H., Zou L. (2019). Tai Chi as an alternative exercise to improve physical fitness for children and adolescents with intellectual disability. Int. J. Environ. Res. Public Health.

[33] Milanovic I., Radisavljevic-Janic S., Zivkovic M.Z., Mirkov D.M. (2019). Health-related physical fitness levels and prevalence of obesity in Serbian elementary schoolchildren. Nutr. Hosp..

[34] Alcántara-Cordero F.J., Gómez-Píriz P.T., Sánchez-López A.M., Cabeza-Ruiz R. (2020). Feasibility and reliability of a physical fitness tests battery for adults with intellectual disabilities: The SAMU DIS-FIT battery. Disabil. Health J..

[35] Daniel M.V., Rafael M.M., Jesús V. (2014). Criterion-related validity of sit-and-reach tests for estimating hamstring and lumbar extensibility: A meta-analysis. J. Sports Sci. Med..

[36] Hsieh K., Rimmer J.H., Heller T. (2014). Obesity and associated factors in adults with intellectual disability. J. Intellect. Disabil. Res..

[37] Bennett E.A., Kolko R.P., Chia L., Elliott J.P., Kalarchian M.A. (2017). Treatment of obesity among youth with intellectual and developmental disabilities: An emerging role for telenursing. West J. Nurs. Res..

[38] Phillips K.L., Schieve L.A., Visser S., Boulet S., Sharma A.J., Kogan M.D., Boyle C.A., Yeargin-Allsopp M. (2014). Prevalence and impact of unhealthy weight in a national sample of US adolescents with autism and other learning and behavioral disabilities. Matern. Child Health J..

[39] Chen A.Y., Kim S.E., Houtrow A.J., Newacheck P.W. (2010). Prevalence of obesity among children with chronic conditions. Obesity.

[40] Westrop S.C., Melville C.A., Muirhead F., McGarty A.M. (2019). Gender differences in physical activity and sedentary behaviour in adults with intellectual disabilities: A systematic review and meta-analysis. J. Appl. Res. Intellect. Disabil..

[41] Gonzalez-Suarez C.B., Grimmer-Somers K. (2011). The association of physical activity and physical fitness with pre-adolescent obesity: An observational study in Metromanila, Philippines. J. Phys. Act. Health.

[42] Ortega F.B., Ruiz J.R., Hurtig-Wennlöf A., Vicente-Rodríguez G., Rizzo N.S., Castillo M.J., Sjöström M. (2010). Cardiovascular fitness modifies the associations between physical activity and abdominal adiposity in children and adolescents: The European Youth Heart Study. Br. J. Sports Med..

[43] Lee Y.G., Oh S.H. (2012). The Relationship of obesity to health-related physical fitness of secondary school boys and girls a study on. Korean J. Meas. Eval. Phys. Educ. Sports Sci..

[44] ACSM (2017). ACSM’s Guidelines for Exercise Testing and Prescription.

[45] Garber C.E., Blissmer B., Deschenes M.R., Franklin B.A., Lamonte M.J., Lee I.-M., Nieman D.C., Swain D.P. (2011). Quantity and quality of exercise for developing and maintaining cardiorespiratory, musculoskeletal, and neuromotor fitness in apparently Healthy Adults: Guidance for Prescribing Exercise. Med. Sci. Sports Exerc..

[46] Nelson M.E., Rejeski W.J., Blair S.N., Duncan P.W., Judge J.O., King A.C., Macera C.A., Castaneda-Sceppa C. (2007). Physical activity and public health in older adults: Recommendation from the American College of Sports Medicine and the American Heart Association. Med. Sci. Sports Exerc..

[47] Fernhall B., Mendonca G.V., Baynard T. (2013). reduced work capacity in individuals with down syndrome: A consequence of autonomic dysfunction?. Exerc. Sport Sci. Rev..

[48] López-Gil J.F., Brazo-Sayavera J., Yuste Lucas J.L., Renato Cavichiolli F. (2020). Weight status is related to health-related physical fitness and physical activity but not to sedentary behaviour in children. Int. J. Environ. Res. Public Health.

[49] Miyatake N., Miyachi M., Tabata I., Sakano N., Hirao T., Numata T. (2012). Relationship between muscle strength and anthropometric, body composition parameters in Japanese adolescents. Health.

[50] Siff M., Zatsiorsky V. (2001). Biomechanical foundations of strength and power training. Biomechanics in Sport.

[51] Stone M.H. (1993). Position statement: Explosive exercises and training. Natl. Strength Cond. Assoc. J..

[52] Suchomel T.J., Nimphius S., Stone M.H. (2016). The importance of muscular strength in athletic performance. Sports Med..

[53] Shields N., Taylor N.F., Fernhall B. (2010). A study protocol of a randomised controlled trial to investigate if a community based strength training programme improves work task performance in young adults with Down syndrome. BMC Pediatr..

[54] Xu C., Yao M., Kang M., Duan G. (2020). Improving Physical Fitness of Children with Intellectual and Developmental Disabilities through an Adapted Rhythmic Gymnastics Program in China. BioMed Res. Int..

[55] Benetti F.A., Bacha I.L., Junior A.B.G., Greve J.M.D. (2016). Analyses of balance and flexibility of obese patients undergoing bariatric surgery. Clinics.

[56] Oliveira A., Monteiro Â., Jácome C., Afreixo V., Marques A. (2017). Effects of group sports on health-related physical fitness of overweight youth: A systematic review and meta-analysis. Scand. J. Med. Sci. Sports.

[57] Shore S.M., Sachs M.L., DuCette J.P., Libonati J.R. (2014). Step-count promotion through a school-based intervention. Clin. Nurs. Res..

[58] Nahas M.V. (2003). Atividade Física, Saúde e Qualidade de Vida: Conceitos e Sugestões para um Estilo de Vida Ativo.

